# A potential role for SARS-CoV-2 small viral RNAs in targeting host microRNAs and modulating gene expression

**DOI:** 10.1038/s41598-022-26135-9

**Published:** 2022-12-15

**Authors:** Zachary T. Neeb, Alexander J. Ritter, Lokendra V. Chauhan, Sol Katzman, W. Ian Lipkin, Nischay Mishra, Jeremy R. Sanford

**Affiliations:** 1 Department of Molecular, Cell and Developmental Biology and Center for Molecular Biology of RNA, University of California Santa Cruz, Santa Cruz, CA USA; 2 Department of Biomolecular Engineering, University of California Santa Cruz, Santa Cruz, CA USA; 3 Center for Infection and Immunity, Mailman School of Public Health, Columbia University, New York, NY USA; 4 UC Santa Cruz Genomics Institute, University of California Santa Cruz, Santa Cruz, CA USA

**Keywords:** SARS-CoV-2, miRNAs, Small RNAs, Non-coding RNAs, RNA metabolism

## Abstract

Severe acute respiratory syndrome coronavirus 2 (SARS-CoV-2) causes coronavirus disease (COVID-19) in humans, with symptoms ranging from mild to severe, including fatality. The molecular mechanisms surrounding the effects of viral infection on the host RNA machinery remain poorly characterized. We used a comparative transcriptomics approach to investigate the effects of SARS-CoV-2 infection on the host mRNA and sRNA expression machinery in a human lung epithelial cell line (Calu-3) and an African green monkey kidney cell line (Vero-E6). Upon infection, we observed global changes in host gene expression and differential expression of dozens of host miRNAs, many with known links to viral infection and immune response. Additionally, we discovered an expanded landscape of more than a hundred SARS-CoV-2-derived small viral RNAs (svRNAs) predicted to interact with differentially expressed host mRNAs and miRNAs. svRNAs are derived from distinct regions of the viral genome and sequence signatures suggest they are produced by a non-canonical biogenesis pathway. 52 of the 67 svRNAs identified in Calu-3 cells are predicted to interact with differentially expressed miRNAs, with many svRNAs having multiple targets. Accordingly, we speculate that these svRNAs may play a role in SARS-CoV-2 propagation by modulating post-transcriptional gene regulation, and that methods for antagonizing them may have therapeutic value.

## Introduction

Small non-coding RNAs (sRNAs) play diverse roles in gene regulation and genome integrity. Ranging from ~ 20–30 nt, this functionally diverse class of RNAs plays important roles in the regulation of many biological processes^1,2^. Short interfering RNAs and microRNAs (siRNA and miRNA, respectively) function in post-transcriptional control of gene expression by regulating messenger RNA (mRNA) translation and stability^3,4^. By contrast, Piwi-associated RNAs (piRNAs) control transcriptional silencing of transposable elements in eukaryotic germlines and the elimination of entire regions of ciliate genomes^5,6^.

sRNAs are derived from larger precursor transcripts. Although biogenesis pathways for the different types of sRNAs vary widely depending on type and species, processing of precursors typically involves cleavage by the endonucleases Drosha and Dicer before they are loaded onto an Argonaute Family protein (either an AGO or a PIWI) to interact with downstream targets. However, there are a multitude of alternative mechanisms for sRNA biogenesis, some of which include Drosha/Dicer-independent mechanisms or even multiple Argonautes and Dicer-like proteins^7–10^. The level of complementarity of an sRNA to its target varies from as little as 8 nt in the 5ʹ seed region (miRNAs) up to 100% complementarity of the full sRNA sequence (siRNAs and piRNAs), and often is a determining factor in how an sRNA interacts with its targets. It has also been suggested for human miRNAs that their abundance directly affects whether they repress translation by active mRNA degradation, or by interaction with the 3ʹ UTR when miRNA levels are insufficient for widespread 3ʹ UTR binding^11^. Taken as a whole, small RNA biogenesis and function is complex and exceptions to rules regarding roles and mechanisms are quite common.

Viruses such as SARS-CoV-2 remodel host gene expression programs. This occurs through a variety of mechanisms, including deregulation of post-transcriptional gene regulation. More recently, viral small non-coding RNAs have also been identified that may play important roles in adaptation and viral propagation^12–15^ in infections with SARS-CoV-1^12^, Influenza^13^, Hepatitis A^14^ and EV71^15^. During the COVID-19 pandemic, multiple groups also reported the existence of miRNA-like sRNAs derived from SARS-CoV-2, although the details of their biogenesis and specific function have yet to be elucidated^16–18^. Small viral RNAs (svRNAs) vary greatly in size and cellular abundance and have been implicated in a variety of different biological pathways related to infection and host immune evasion^19^.

In this study, we characterize global changes in both host cell mRNA and miRNA expression using multiple sequencing-based methods taking a comparative transcriptomics approach in two very well-established primate cell lines^20–23^. This approach enables identification of conserved changes in host gene regulatory reponses during infection. We also report the discovery of a diverse landscape of svRNAs produced by SARS-CoV-2. Sequence alignments, along with expression dynamics suggest the intriguing hypothesis that SARS-CoV-2 small RNAs may directly regulate host transcripts, including microRNAs.

## Results

### SARS-CoV-2 infection induces global changes in host gene expression

**Figure 1:**
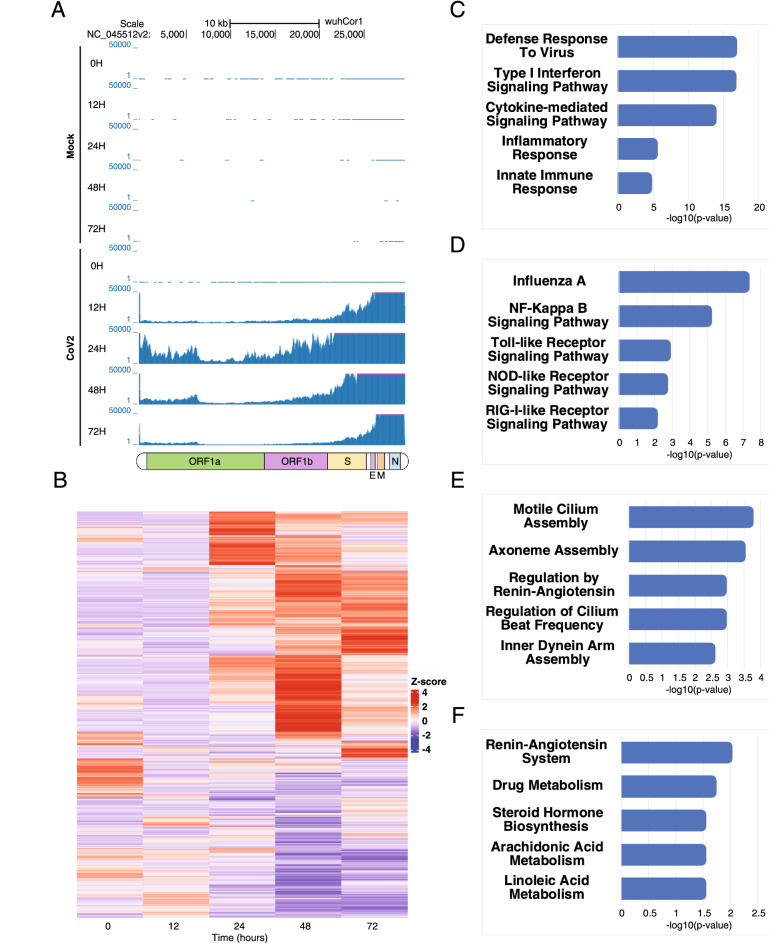
SARS-CoV-2 infection induces global changes in gene expression. (**A**) UCSC Genome Browser screenshots showing Calu-3 sample-derived mRNA sequencing reads mapped to the SARS-CoV-2 genome for infected and control cells. Underneath the screenshot is a schematic representation of the SARS-CoV-2 genome depicting the regions of ORF1a/1b, the spike protein (S), the envelope protein (E), the membrane protein (M) and the nucleocapsid protein (N). (**B**) Heatmap representation of differentially expressed Calu-3 genes over the time course of infection. 4593 differentially expressed genes are shown. Heatmaps were generated using hierarchical clustering. (**C**) Enriched Gene Ontology Biological Process (GOBP) terms are shown for upregulated Calu-3 genes. Only a subset of significantly enriched GOBP terms are shown. (**D**) Same as (**C**), but with enriched KEGG Human Pathways. (**E**) Enriched GOBP terms for downregulated Calu-3 genes. Only a subset of significantly enriched GOBP terms are shown. (**F**) Same as (**E**), but with enriched KEGG Human Pathways.

To investigate how SARS-CoV-2 infection influences host gene expression, we analyzed the transcriptomes of both a human lung epithelial cell line (Calu-3) and African green monkey kidney cell line (Vero-E6) during the course of SARS-CoV-2 infection. Both cell lines were infected with SARS-CoV-2 at a multiplicity of infection (MOI) of 0.01. RNA extracts were made from harvested cell pellets of infected Calu-3 cells at 0 h, 12 h, 24 h, 48 h and 72 h post-infection (hpi), and infected Vero-E6 cells at 4 h, 24 h, 48 h, 72 h, 120 h, 165 h and 216 hpi. RNA extracts of cell pellets from uninfected cells were also used as controls and processed from all time points. Similar to previous studies^24^, the majority of reads from infected cells mapped to the host genomes (60.3–100.0% for Calu-3 cells, 88.2–95.6% for Vero-E6 cells), with the remainder mapping to the SARS-CoV-2 genome (Fig. 1A, Supplemental Table S1).

SARS-CoV-2 infection induces global changes in gene expression in both Calu-3 cells and Vero-E6 cells. DESeq2^25^ analysis revealed 4,593 and 1,040 significantly differentially expressed genes, respectively (Fig. 1B, Supplemental Fig. S1). Differentially expressed genes in Calu-3 and Vero-E6 cells were used for downstream Gene Ontology Biological Process (GOBP) and KEGG Human Pathway enrichment analysis. Upregulated genes fell into enriched gene families related to innate immunity and inflammatory response pathways, while downregulated genes tended to be involved in metabolic and other biosynthetic pathways (Fig. 1C–F, Supplemental Tables S2-S4). For both Calu-3 and Vero-E6 cells, we observed significant overlap between GOBP terms for upregulated and downregulated genes throughout the time course. By contrast, KEGG pathway overlap was only observed for the upregulated gene sets (Supplemental Fig. S2). Interestingly, for both cell types, we observed gene expression changes to be most significant from 24 to 48 hpi, most likely reflective of the time required to elicit cellular response to viral infection. Overall, we found SARS-CoV-2 infection to cause global gene expression changes in both Calu-3 and Vero-E6 cells.

### SARS-CoV-2 expresses a diverse landscape of small RNAs

**Figure 2:**
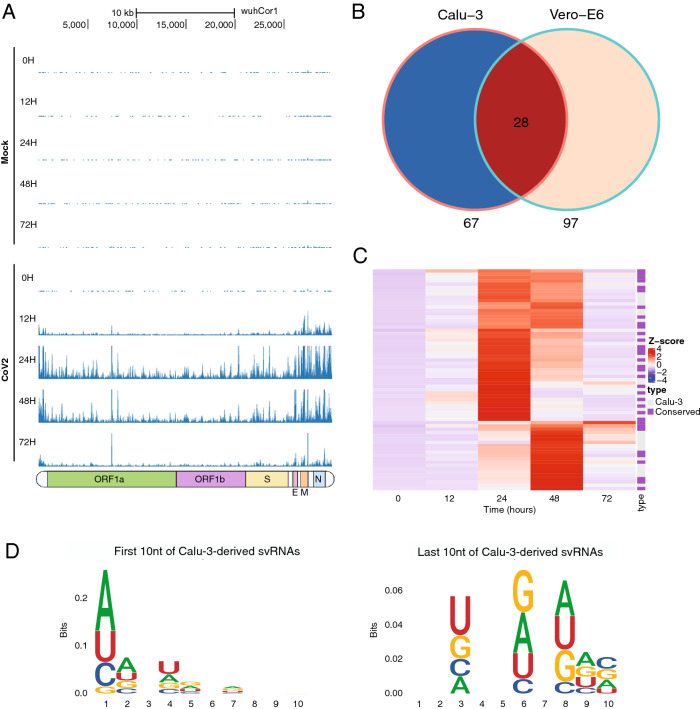
SARS-CoV-2 expresses a diverse landscape of small RNAs. (**A**) UCSC Genome Browser screenshots showing Calu-3 sample-derived sRNA sequencing reads mapped to the SARS-CoV-2 genome for both infected and control cells. Reads from infected cells do not map uniformly to the genome but instead form “pile ups” in particular regions. Underneath the screenshot is a schematic representation of the SARS-CoV-2 genome depicting the regions of ORF1a/1b, the spike protein (S), the envelope protein (E), the membrane protein (M) and the nucleocapsid protein (N). (**B**) Venn Diagram showing the number of svRNAs identified in Calu-3 and Vero-E6 cells, with conserved loci indicated. (**C**) Heatmap representation of Calu-3 cell-derived svRNA expression. Heatmaps were generated using hierarchical clustering. To the right of the heatmap is an annotation column indicating if the svRNA is species-specific or conserved between Calu-3 and Vero-E6 cells. (**D**) Sequence logos for the first ten and last ten nucleotides of the svRNAs identified in Calu-3 cells. svRNAs lack a 5’-U signature typical of many canonical miRNAs.

**Figure 3:**
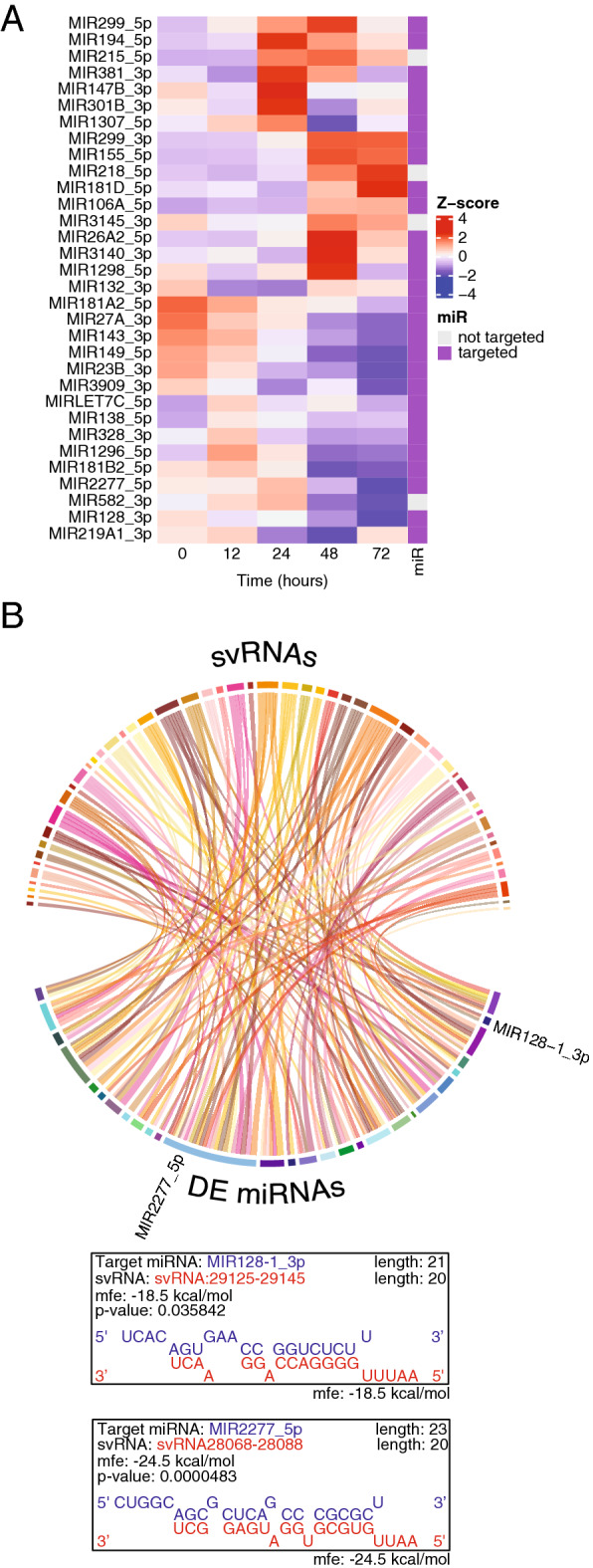
SARS-CoV-2 svRNAs may target host miRNAs. (**A**) Heatmap representation of the 28 differentially expressed Calu-3 miRNAs over the time course of infection. Heatmaps were generated using hierarchical clustering. To the right of the heatmap is an annotation column indicating if the miRNA is a predicted target of an svRNA. (**B**) Circos plot representation of predicted interactions between svRNAs and DE human miRNAs using RNAhybrid without seed-forcing. The ribbons are colored according to the svRNA that is predicted to interact with the miRNA to which it connects. There are many examples of svRNAs targeting more than one miRNA and of miRNAs being targeted by more than one svRNA. DE miRNAs mentioned in the Discussion section are labeled. Shown below are two examples of RNAhybrid prediction outputs for svRNAs and DE miRNAs pictured above.

We analyzed the small RNA transcriptome from mock and infected Calu-3 and Vero-E6 cells to uncover potential SARS-CoV-2-dependent gene regulatory mechanisms. Following infection, libraries from both cell lines contained reads mapping to the SARS-CoV-2 genome (Fig. 2A, Supplemental Fig. S3, Supplemental Table S1). We found that the distribution of svRNAs mapping to the SARS-CoV-2 genome was not uniform and that there were “pile-ups'' in particular regions indicating the RNAs identified are not likely to be degradation products of the full-length viral genomic or subgenomic RNA, but may be derived from specific loci (Fig. 2A, Supplemental Fig. S3). Using Piranha^26^, we identified 67 svRNAs from Calu-3 cells with a mean length of 22 nt and 97 svRNAs from Vero-E6 cells with a mean length of 25 nt (Fig. 2B, Supplemental Figs. S4, S5, Supplemental Table S3). 28 svRNA loci were shared between the two cell lines (Fig. 2B). svRNA expression is dynamic throughout the time course with peak expression of the svRNAs between 24 and 48 hpi for both species (Fig. 2C, Supplemental Fig. S6). Calu-3 svRNAs tend to cluster into three distinct types of expression profiles. Two svRNA groups showed transient peak expression at 24 and 48 hpi respectively and then returned to base-level, while another group showed sustained expression from 24 to 48 hpi (Fig. 2C). The dynamics of svRNA expression follows similar kinetics as changes in host mRNA and miRNA expression (Figs. 1B, 3A, Supplemental Fig. S1). To assess the conservation of svRNAs among variants of SARS-CoV-2 we intersected them with known mutations in variants of concern from the Uniprot Amino Acid mutations track on the UCSC Genome Browser. While 6 of the 67 Calu-3-specific svRNAs and 5 of the 66 conserved svRNAs overlapped with at least one mutation, only 3 and 4 of them respectively overlap in such a way that the seed sequence would be affected (Supplemental Table S8, Supplemental Fig. S14). We therefore speculate that these mutations would not significantly impact svRNA function. Further studies are needed to answer this question and we are highly interested in infecting Calu-3 cells with SARS-CoV-2 variants in the future to investigate the functional effects of these mutations.

To illuminate potential biogenesis pathways for SARS-CoV-2 svRNAs, we aligned the 5ʹ and 3ʹ ends of svRNAs searching for nucleotide biases. By contrast to miRNAs, the svRNAs did not possess sequence signatures with the typical 5ʹ-U bias, suggesting a Dicer-independent processing pathway (Fig. 2D, Supplemental Fig. S7). Additionally, we found that only 10 of the 67 Calu-3 cell-derived svRNAs and 12 of the 97 Vero-E6 cell-derived svRNAs were predicted to have an upstream or downstream complementary sequence within 44 nt that may form a hairpin^27^, often required for canonical miRNA processing. (Supplemental Table S6).

### SARS-CoV-2 alters host microRNA expression

We hypothesized that global changes in gene expression could be mediated through changes in host sRNA expression, specifically miRNAs. We analyzed the host small RNA transcriptomes of infected and control Calu-3 cells (Fig. 3A, Supplemental Figs. S8–S10, Supplemental Table S1) and discovered that 28 miRNAs are differentially expressed throughout the infection time course (Fig. 3A, Supplemental Table S7). Consistent with our mRNA sequencing data, we observed differential expression of miRNAs at 24 hpi with the most significant changes occurring at 48 hpi (Fig. 1B).

### SARS-CoV-2 svRNAs may hybridize with differentially expressed mRNAs and miRNAs

**Figure 4:**
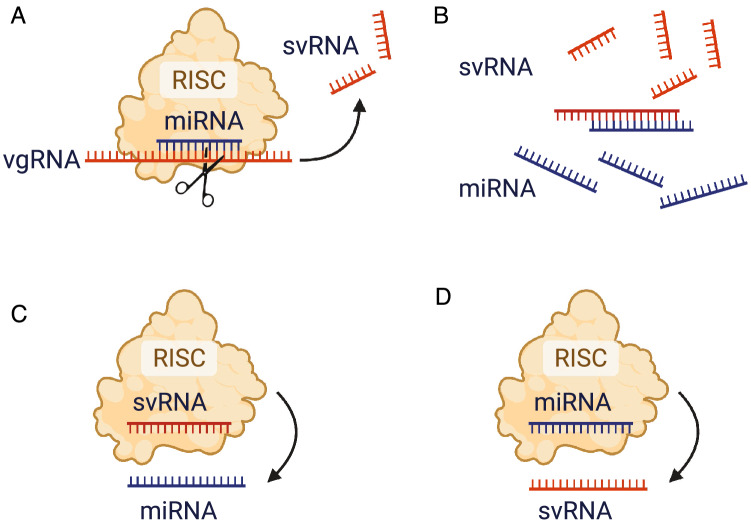
Proposed svRNA-miRNA interactions upon SARS-CoV-2 infection. The illustrated model is briefly described in the Discussion section. svRNAs may interact with miRNAs in multiple ways, none of which are mutually exclusive. (**A**) A RISC-miRNA complex targets complementary sequences in the viral genomic RNA. svRNAs are produced by cleavage and go on to perform downstream functions, such as interacting with host mRNAs and miRNAs. (**B**) There is direct hybridization of svRNAs and miRNAs without association with RISC, leading to sequestration of miRNAs, preventing them from interacting with endogenous targets. (**C**) A RISC-svRNA complex targets miRNAs and prevents them from interacting with endogenous targets. (**D**) A RISC-miRNA complex targets svRNAs, preventing the complex from interacting with endogenous targets. Created with BioRender.com.

To investigate the potential function of svRNAs we used RNAHybrid^28,29^ to identify targets within differentially expressed Calu-3 mRNAs and miRNAs. Of the svRNAs identified in Calu-3 cells, 59 of 67 have predicted mRNA targets within the 3’UTRs of differentially expressed (DE) genes (Tables 1 and 2, Supplemental Tables S2-3). RNAhybrid predictions identified svRNAs that can also form stable duplexes with miRNAs, 52 of which target differentially expressed miRNAs in our data set (Fig. 3B, Table 3, Supplemental Fig. S11, Supplemental Table S7). A larger portion of svRNA-target interactions were predicted to occur with downregulated miRNAs than with downregulated mRNA targets throughout the time course (Supplemental Fig. S10). Interestingly, we found numerous examples of host miRNAs that can pair with multiple svRNAs (Figs. 3B, 4, Supplemental Fig. S11). In addition, we also observed examples of svRNAs that can potentially hybridize to a broad array of miRNAs (Figs. 3B, 4, Table 3, Supplemental Fig. S11). Because the Vero-E6 genome has not been extensively annotated to include miRNA sequences, we were unable to perform RNAhybrid predictions against Vero-E6 miRNAs, but found that of the 61 Calu-3-derived svRNAs capable of targeting host miRNAs, 27 had conserved loci with Vero-E6-derived svRNAs.

**Table 1:** List of the ten most abundant Calu-3-derived svRNAs with DE mRNA targets.

svRNA	Counts	# mRNA targets	Top enriched GO term for targets
svRNA2875-2897	165	39	Negative regulation of CD4-positive, alpha–beta T cell proliferation (GO:2000562)
svRNA26927-26947	134	18	Negative regulation of CD4-positive, alpha–beta T Cell proliferation (GO:2000562)
svRNA28260-28289	89	1	Gamma-aminobutyric acid metabolic process (GO:0009448)
svRNA27059-27089	88	16	Regulation of protein import into nucleus (GO:0042306)
svRNA27107-27128	80	11	Heart field specification (GO:0003128)
svRNA26820-26840	77	2	Mesoderm morphogenesis (GO:0048332)
svRNA5572-5597	64	4	Regulation of oxidoreductase activity (GO:0051341)
svRNA37-63	57	2	Ribosomal Large Subunit Assembly (GO:0000027)
svRNA18943-18965	47	1	N/A
svRNA29039-29064	45	5	Extracellular structure organization (GO:0043062)

Table listing the ten most abundant svRNAs with DE mRNA targets, normalized counts, number of differentially expressed mRNA targets and the top enriched GO term for targets. Counts were normalized to the mean read depth of all sample libraries.

**Table 2:** List of the top enriched GO terms for DE mRNA targets of Calu-3-derived svRNAs.

Top enriched GO terms for downregulated mRNA targets of svRNAs	Top enriched GO terms for upregulated mRNA targets of svRNAs
Quinone catabolic process (GO:1901662)	Cellular response to cytokine stimulus (GO:0071345)
Leukotriene B4 metabolic process (GO:0036102)	Cytokine-mediated signaling pathway (GO:0019221)
Menaquinone metabolic process (GO:0009233)	Regulation of interferon-gamma production (GO:0032649)
Vitamin K metabolic process (GO:0042373)	Cellular response to interferon-gamma (GO:0071346)
Fat-soluble vitamin metabolic process (GO:0006775)	Negative regulation of natural killer cell mediated cytotoxicity (GO:0045953)
Organic hydroxy compound catabolic process (GO:1901616)	Regulation of apoptotic cell clearance (GO:2000425)
Fat-soluble vitamin catabolic process (GO:0042363)	Positive regulation of apoptotic cell clearance (GO:2000427)
Axoneme assembly (GO:0035082)	interferon-gamma-mediated Signaling Pathway (GO:0060333)
Diterpenoid metabolic process (GO:0016101)	Regulation of immune response (GO:0050776)
Phosphate ion transport (GO:0006817)	Negative regulation of natural killer cell mediated immunity (GO:0002716)

Table listing the top ten significantly enriched GO terms for all upregulated and all downregulated mRNA targets of Calu-3-derived svRNAs in our data set.

**Table 3:** List of the ten most abundant Calu-3-derived svRNAs with DE miRNA targets.

svRNA	Counts	# miRNA targets	miRNA
svRNA26741-26761	165	4	**MIR128_3p**, MIR1298_5p, **MIR1307_5p**, **MIR2277_5p**
svRNA28573-28595	134	1	**MIR2277_5p**
svRNA2868-2897	88	3	**MIR106A_5p**, **MIR2277_5p**, MIR381_3p
svRNA28704-28725	80	2	**MIR138_5p**, **MIR2277_5p**
svRNA28553-28573	77	4	**MIR132_3p**, **MIR2277_5p**, **MIR23B_3p**, MIR299_5p
svRNA18943-18965	57	1	**MIR149_5p**
svRNA27578-27602	47	2	MIR26A2_5p, MIR381_3p
svRNA524-546	45	1	**MIR2277_5p**
svRNA5423-5445	45	3	MIR1298_5p, **MIR181B2_5p, MIR3909_3p**
svRNA28068-28088	41	1	**MIR132_3p**

Table listing the ten most abundant svRNAs with DE miRNA targets, normalized counts, number of differentially expressed miRNA targets and miRNA target names. miRNAs in bold are downregulated in our data set. Counts were normalized to the mean read depth of all sample libraries.

## Discussion

We analyzed the transcriptomes of two primate cell lines during SARS-CoV-2 infection and observed global changes in gene expression. Consistent with previous work, we found enrichment for upregulated genes involved in the RIG-I pathway, Toll-like Receptor (TLR) signaling pathways, NF-kappa Beta pathway, and type 1 interferon (IFN) signaling^16,30–34^ (Supplemental Fig. S12). Downregulated genes tended to fall into enriched gene families involved with metabolic and other biosynthetic pathways, consistent with SARS-CoV-2-mediated metabolic dysregulation observed in human patients^35–37^. Metabolic dysregulation may lead to an increase in the inflammatory and auto-immune responses as observed in severe Covid-19 patients. We also identified more than a hundred svRNAs capable of interacting with host transcripts, including miRNAs. svRNAs are expressed from specific loci, within ORF7a, and the spike (S) and nucleocapsid (N) genes. In addition to having predicted target sites in differentially expressed host transcript 3’-UTRs like canonical miRNAs, 52 of the 67 Calu-3 sample-derived svRNAs are also predicted to form stable duplexes with differentially expressed miRNAs, many of which have been implicated in other types of viral respiratory infections (VRIs)^38^. For example, miR-128, miR-2277 and miR-155 are known to be associated with rhinovirus infection, Middle Eastern Respiratory Syndrome (MERS) and SARS-CoV-2, respectively. We see many examples of svRNAs with predicted target sites in more than one differentially expressed miRNA, with 38 of the 67 having two or more predicted targets. svRNAs are most highly expressed at 24 h and 48 hpi, which corresponds to the time points at which we see the most significant changes in host gene and miRNA expression. This expression dynamic suggests an interplay between svRNAs and host miRNAs, in which svRNAs have the capability to interact with host miRNAs, therefore affecting downstream target mRNA expression.

Here we describe an expanded landscape of SARS-CoV-2-derived small RNAs including all four svRNAs previously reported by the Cheng, Meng and Pawlica groups^16–18^ (Supplemental Fig. S13). In agreement with what the Meng and Cheng groups observed, we identified dozens of svRNAs derived from the nucleocapsid (N) gene, many that overlap the sequences reported in those studies, albeit with varying starting coordinates and lengths^16,17^ (Supplemental Fig. S13). In addition to recapitulating the existence of svRNAs derived from the N gene, we identified an svRNA (svRNA27406-27428) derived from ORF7a that almost perfectly overlaps with the sequence reported by Pawlica and colleagues^18^ (Supplemental Fig. S13). We reason that our experiment has increased sensitivity because we use a lower MOI (0.01) than previous studies (0.05–5), and performed an extended time course experiment^16,18^. In contrast to previous studies, we have shown SARS-CoV-2 infection to cause significant changes in global host miRNA expression and have additionally predicted the svRNAs identified here to interact with differentially expressed host miRNAs.

miRNAs are typically processed via a Dicer-mediated biogenesis pathway. Our data suggest that many of the svRNAs are generated instead via a Dicer-independent processing mechanism. Lack of a 5ʹ-U bias in the sequence analyses for svRNAs indicates that biogenesis may occur in a non-canonical Dicer-independent manner. Other groups have also reported svRNAs derived from SARS viruses lacking a 5ʹ-U sequence signature and it has been suggested that these small RNAs may utilize the slicer activity of Ago2 for biogenesis rather than the traditional miRNA/siRNA processing machinery, similar to what has been observed for the well-studied human miR-451^10,12,17^. Morales and colleagues observed svRNA expression following knockdown of the RNase III nucleases Drosha and Dicer^12^, suggesting these canonical enzymes are not necessary for svRNA biogenesis. We also identified svRNAs derived from ORF7a and the N gene that overlap with svRNAs reported by the Meng, Cheng and Pawlica groups, but with varied starting coordinates and/or sizes. This may suggest that isomiRs exist for svRNAs, potentially caused by alternative processing events such as 5ʹ/3ʹ trimming variants due to imprecise cleavage of precursors^39^. Further studies on svRNAs derived from SARS-CoV-2 are necessary to elucidate key players in their biogenesis and to validate their potential to interact with host transcripts to affect host gene expression.

Based on our observations, we propose a functional role for svRNAs in which deregulation of the host mRNA expression program may be a direct effect of svRNAs sequestering or “sponging” host miRNAs (Fig. 4). There are many examples of known miRNA sponges in the literature, such as *HSUR 1* and *HSUR 2* from *Herpesvirus saimiri *^40^, and it is entirely possible that the svRNAs presented here may function similarly^41–43^. An efficient way to post-transcriptionally alter a gene network or biological system would be to sequester/act on regulators of the network itself, rather than individual genes separately. As canonical human miRNAs often have hundreds of predicted target genes each, production of svRNAs that interfere with this regulation may confer an evolutionary advantage for SARS-CoV-2 and other viruses by modulating host gene expression on a global scale, increasing the likelihood of viral propagation. It has been previously suggested that viral RNA derived from SARS-CoV-2 may act as a host miRNA sponge to aid in evading the host immune response and our findings add more credence to this hypothesis^44^. Based on RNAhybrid seed-target predictions, 22 of 33 differentially expressed host miRNAs are capable of targeting svRNAs, 11 of which are upregulated during the time course. It is possible that in addition to svRNAs sponging host miRNAs, they also directly target miRNAs with or without RISC, preventing the host miRNAs from interacting with endogenous targets (Fig. 4). These potential svRNA-miRNA interactions are not mutually exclusive and may occur simultaneously, ultimately leading to the same outcome: global changes in host gene expression. Direct interactions between svRNAs and their predicted targets must still be validated experimentally to assess the full extent to which these interactions occur during viral infection and also to tease apart the actual targeting mechanism itself. In the future, these interactions could be easily antagonized or blocked using locked nucleic acids (LNAs) or other methods and potentially used as targets for the therapeutic treatment of patients infected with the virus.

## STAR methods

### Cell culture and viruses

African green monkey kidney (Vero-E6) cells and human lung epithelial (Calu-3) cells were obtained from the ATCC (Manassas, VA). The cells were cultured in Dulbecco’s modified Eagle’s medium (ThermoFisher Scientific, Waltham, MA, USA) containing 1% heat inactivated fetal bovine serum (ThermoFisher Scientific), 100 U/mL penicillin, and 100 μg/mL streptomycin. The cells were incubated in 95% air and 5% CO_2_ at 37 °C.

### Virus infection and total RNA extraction

Vero-E6 and Calu-3 cells (2 × 10^5^ cells/well in 6-well plates) were cultured in DMEM containing 1% FBS at 37 °C in a CO_2_ incubator overnight. The cells were washed once with phosphate-buffered saline (PBS), prototypic SARS-CoV-2 Washington strain (USA/WA1/2020) in growth media at MOI 0.01 was added into each desired well, and the plates were incubated for 1.5 h at 37 °C in a CO_2_ incubator. After incubation, 3 mL of DMEM containing 1% FBS was added to each well and the plates were incubated at 37 °C in a CO_2_ incubator. Infected Calu-3 cells were harvested at 0 h, 12 h, 24 h, 48 h and 72 hpi (in triplicate) and Vero-E6 cells were harvested at 4 h, 24 h, 48 h, 72 h, 120 h, 168 h and 216 hpi (in duplicate).

For harvesting, cell culture supernatants were collected and mixed with Trizol (Fisher Scientific) at a ratio of 1:1. Cells were detached using Trypsin–EDTA 0.25% (ThermoFIsher Scientific), followed by centrifugation at 1000 rpm for 5 min at 4 °C to form a cell pellet which in turn was dissolved in 250 μl of Trizol. Both cell supernatant in Trizol and cell pellet in Trizol were stored at – 80 °C until further processing. SARS-CoV-2 amplification and cell culture procedures were performed according to biosafety level 3 (BSL-3) conditions.

Total RNA was extracted using the TRI Reagent protocol for isolation of RNA with the following modification: one additional RNA ethanol wash step was included. After the total RNA was solubilized in ddH_2_0, one overnight ethanol precipitation step was included for further purification of the total RNA.

### Illumina sequencing of sRNA libraries

Total RNA was isolated from cell culture pellets as described above. Total RNA from infected Calu-3 cells, infected Vero-E6 cells and uninfected controls were used for sRNA library preparation. 1 μg of total RNA for each sample was used for sRNA library preparation using the NEXTFLEX small RNA-Seq Kit v3 following the manufacturer’s protocol (Perkin Elmer Applied Genomics). Adapter dilution was not performed and 17 cycles of PCR amplification were used before using the gel-free size selection and cleanup protocol. Pooled sRNA sequencing libraries were sequenced on an Illumina HiSeq 4000 at the UC Davis Sequencing Core Facility, generating 100 bp single-end reads.

### Illumina sequencing of mRNA libraries

Total RNA was isolated for cell culture pellets as described above. Total RNA from infected Calu-3 cells, infected Vero-E6 cells and uninfected controls were used for mRNA library preparation. 2 μg of total RNA for each sample was used for mRNA library preparation using the NEXTFLEX Rapid Directional RNA-Seq Kit 2.0 following the manufacturer’s protocol (Perkin Elmer Applied Genomics). Before library preparation, total RNA samples were subjected to Poly(A) selection and purification using the NEXTFLEX Poly(A) Beads Kit 2.0 following the manufacturer’s protocol (Perkin Elmer Applied Genomics). Based on sample RNA integrity numbers (RINS) and total RNA input, 9 min fragmentation time was used, adapter concentration was diluted by half and 9 cycles of PCR amplification were used before bead clean up and elution. For Calu-3 cell samples, pooled mRNA sequencing libraries were sequenced on an Illumina NovaSeq S4 at the UC Davis Sequencing Core Facility, generating 150 bp paired-end reads. For Vero-E6 samples, pooled mRNA sequencing libraries were sequenced on an Illumina HiSeq 4000, generating 100 bp single-end reads.

### Analyses of Calu-3 mRNA sequencing data

The full-length paired-end reads were mapped with Bowtie 2^45^ against the set of human repeat-masker elements in the hg38 assembly, as well as a constant poly-A sequence. Reads that were mapped to these elements were removed from further processing. The repeat-filtered full-length reads were mapped with STAR 2.5.3a^46^, using the "–alignEndsType EndToEnd" option, to a single joint target consisting of the hg38 genome assembly plus the wuhCor1 genome. In cases of multiply-mapped reads, only the best mapping was retained. The STAR mapped bam files were divided into separate hg38 and wuhCor1 files for further analysis.

Gene-by-gene coverage was extracted from the hg38 mappings that overlapped any potential exon for each gene in a gene model of hg38. The total coverage for each gene was divided by the total paired-end read length of each mapping to extract read counts as input to DESeq2^25^. DESeq2 was run to compare all replicates of conditions as follows:

1. At each time point, the control vs the infected samples
2. For the controls, comparisons between the time points
3. For the infected samples, comparisons between the time points.

### Analyses of Vero-E6 mRNA sequencing data

The single-end reads were trimmed at the 3ʹ end to leave 50 bp for mapping. The trimmed reads were mapped with Bowtie 2^45^ against a set of repeat elements in the Vero-E6 genome. The trimmed, repeat-filtered and wuhCor1-filtered, single-end reads were mapped with STAR 2.5.3a, using the "–alignEndsType EndToEnd" option, to chlSab2 genome assembly. In cases of multiply-mapped reads, only the best mapping was retained. Potential PCR duplicates (single-end reads mapped to the identical coordinates) were removed from the chlSab2 mappings.

Gene-by-gene coverage was extracted from the chlSab2 mappings that overlapped any potential exon for each gene in a gene model of chlSab2. The total coverage for each gene was divided by the single-end read length of each mapping to extract read counts as input to DESeq2.

DESeq2 was run to compare all replicates of conditions:

1. At each time point, the control vs the infected samples
2. For the controls, comparisons between the time points
3. For the infected samples, comparisons between the time points.

### Gene ontology (GO) and KEGG human pathway analyses

GO process annotations and KEGG human pathway annotations were retrieved using the web tool Enrichr^47–50^. The p-values of enrichment of GO process and KEGG pathways were determined using Enrichr on differentially expressed upregulated and downregulated genes from both Calu-3 and Vero-E6 data sets.

### svRNA discovery from sRNA-seq data

Potential svRNAs were identified from sRNA-seq reads using a stepwise bioinformatic approach which incorporates established tools and bespoke analysis methods. Prerequisite packages include: STAR v2.7.8a^46^, BEDOPS v2.4.40^51^, bedtools v2.28.0^52^, Piranha v1.2.1^26^, Bowtie 2 v2.4.4^45^ and RNAhybrid v2.1.2^29^. The svRNA-discovery pipeline entails the following steps:

1. sRNA-seq libraries were first mapped against their respective host genomes (GRCh38 for human, chlSab2 for African Green Monkey) and against the SARS-CoV-2 genome (wuhCor1) using STAR^53^.
2. Mapping files from the infected samples from Calu-3 and Vero-E6 were subsetted for sequences between 20 and 30 nt in length that mapped to the SARS-CoV-2 genome. Piranha peaks were called for pooled reads from infected samples for each time point in each of the two cell types using bins of size 50 and a background threshold of 0.95.
3. Piranha peaks were pooled for each cell type, and unique reads mapping to them were aligned to their respective host genomes using Bowtie 2 to remove any potential multi-mapping or host-mapped reads from downstream analysis.
4. Of the remaining sequences, those that met our count-based cutoffs were filtered for sequences whose counts across infected replicates and time points were the highest among overlapping sequences. Some overlapping sequences whose counts were especially relatively high were retained. The count-based cutoffs for a given sequence were as follows:
  - It must be present in at least 5 infected samples for Calu-3 and at least 4 for Vero-E6 (due to the difference in replicates).
  - Its total raw counts from infected samples must be at least 20.
  - If present in any control samples, it must not be present in all control samples.
  - If present in any control samples, its total raw counts from control samples must be less than or equal to 10% of the total raw counts from infected samples.
5. Duplexes between host transcripts (all expressed mRNAs and miRNAs) and SARS-CoV-2-derived sequences from Step 4 were then predicted using RNAhybrid^28,29^, which is a tool that is typically used to predict the hybridization, with the smallest minimum free energy, of a short RNA molecule with a longer one. SARS-CoV-2-derived sequences with at least 1 statistically significant (p-value < 0.05) predicted duplex with a host transcript were then considered potential SARS-CoV-2-derived svRNAs. The resultant target predictions were then subsetted for those involving host targets that were significantly differentially expressed (p-value < 0.05, log_2_ fold change ≥ 1.0).
6. Potential svRNAs from both cell types with overlaps to each other (across cell types) greater than or equal to 60% were categorized as svRNAs derived from “conserved loci”. We define conserved loci as loci in the SARS-CoV-2 genome from which potential svRNAs were detected in both the Calu-3 and Vero-E6 experiments. Additionally, overlapping svRNAs within cell type (i.e. shifted 1-2nt down the SARS-CoV-2 genome sequence) that were retained due to their high relative counts were denoted as potential viral isomiRs—in this case being svRNAs from the same approximate locus but with slightly different ends owed to imprecise precursor cleavage.
7. Lastly, to investigate the potential of identified svRNAs to be products of hairpin precursors, we used RNAfold^27^ to predict the structure of each svRNA with the addition of 30–45 nt upstream and downstream separately. In order to call potential hairpin precursors for svRNAs, the structure predictions needed to have: only a single loop—with no shared nucleotides with the mature svRNA sequence, at least 14 base pairings in the stem, and a maximum minimum free energy (MFE) of − 4.3 kcal/mol^54^. Hairpins that passed these filters were visualized using Forna^55^.

## Supplementary Information

Supplementary Information 1.

Supplementary Information 2.

## Data Availability

All RNA-sequencing data can be accessed at the National Center for Biotechnology Institute (NCBI) Gene Expression Omnibus (GEO) database: GSE197521.

## Code availability

No new code was developed for this study.
