# Supplementary material for: A potential role for SARS-CoV-2 small viral RNAs in targeting host microRNAs and modulating gene expression
Source: Sci Rep. 2022 Dec 15;12:21694. doi: 10.1038/s41598-022-26135-9 (PMC9753033; doi:10.1038/s41598-022-26135-9)
Supplement: Supplementary file 1 — Supplementary Information 1. [file 41598_2022_26135_MOESM1_ESM.pdf]

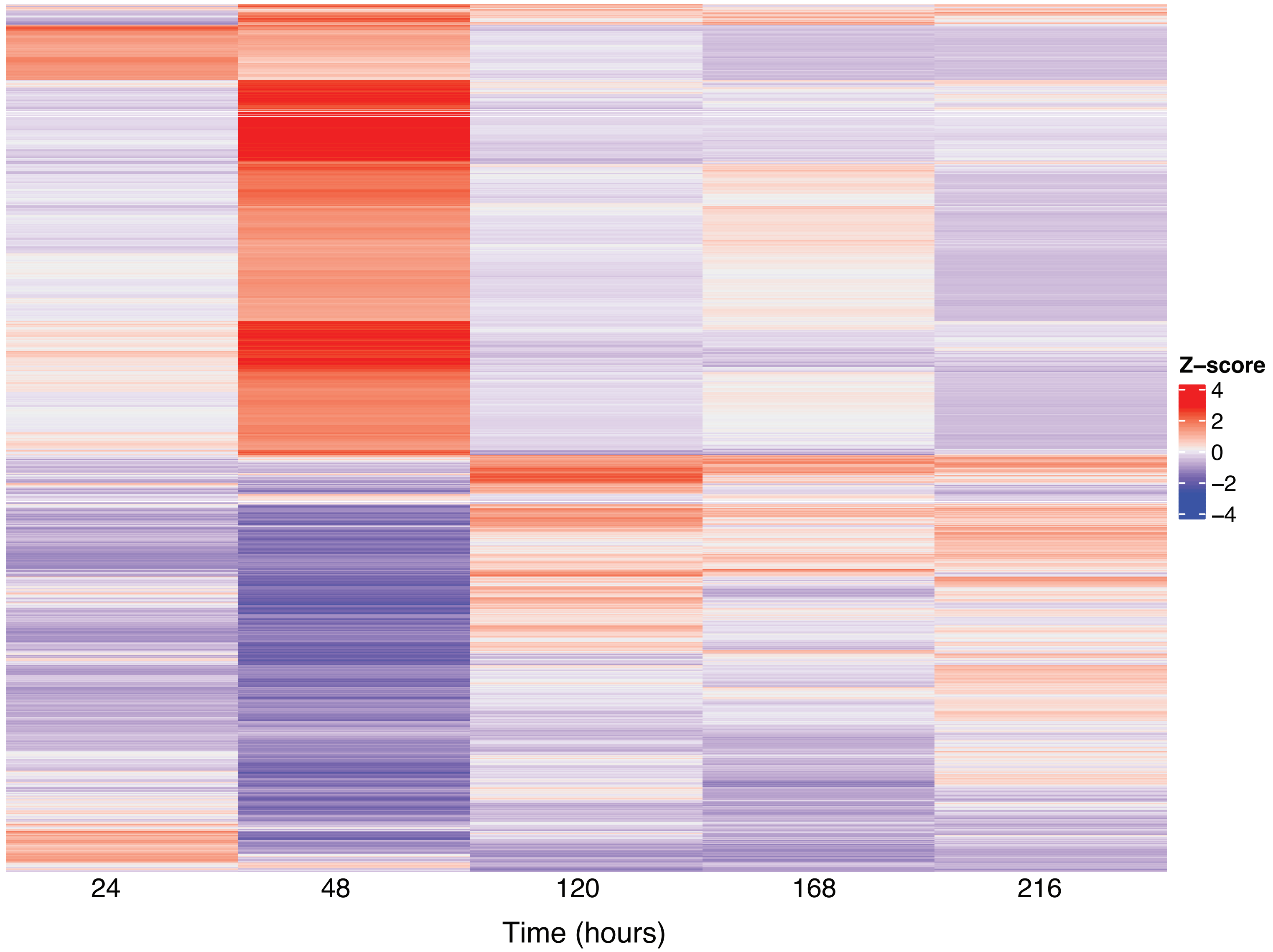

Supplemental Figure 1. SARS-CoV-2 infection induces global changes in gene expression in Vero-E6 cells. Heatmap representation of differentially expressed Vero-E6 genes over the time course of infection. 1,040 differentially expressed genes are shown. Heatmaps were generated using hierarchical clustering. 4 hpi and 72 hpi were omitted for lack of differentially expressed genes during those time points.

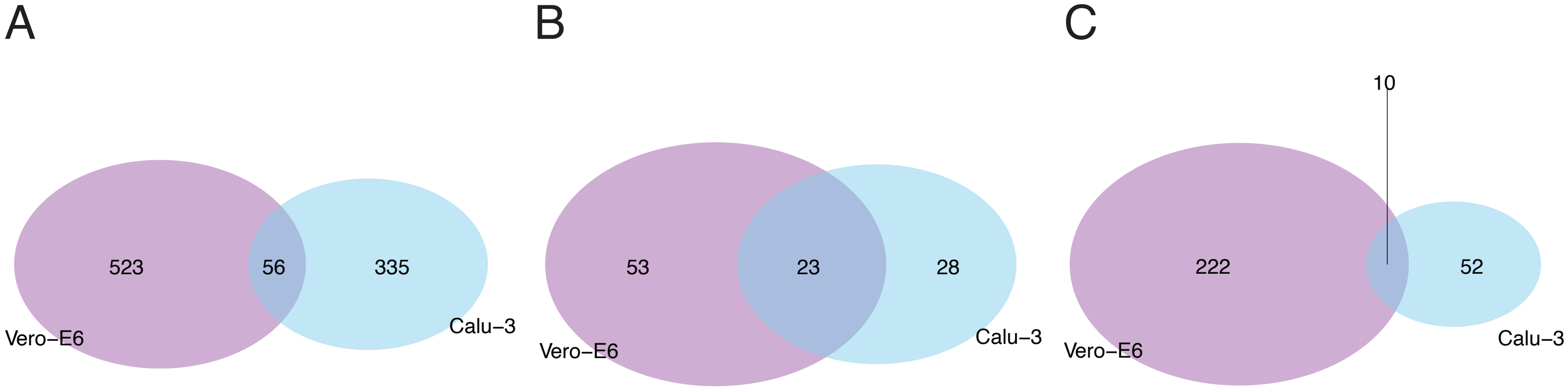

Supplemental Figure 2. Enriched GO Biological Process (GOBP) terms and KEGG human pathways are conserved between infected cell types. A. Venn Diagram depicting the overlap of enriched GOBP terms for upregulated genes in infected Calu-3 and Vero-E6 cells. B. Same as A, but with enriched KEGG Human Pathways. C.Venn Diagram depicting the overlap of enriched GOBP terms for downregulated genes in infected Calu-3 and Vero-E6 cells. There was no overlap between enriched KEGG Human Pathways for downregulated genes between the two cell types.

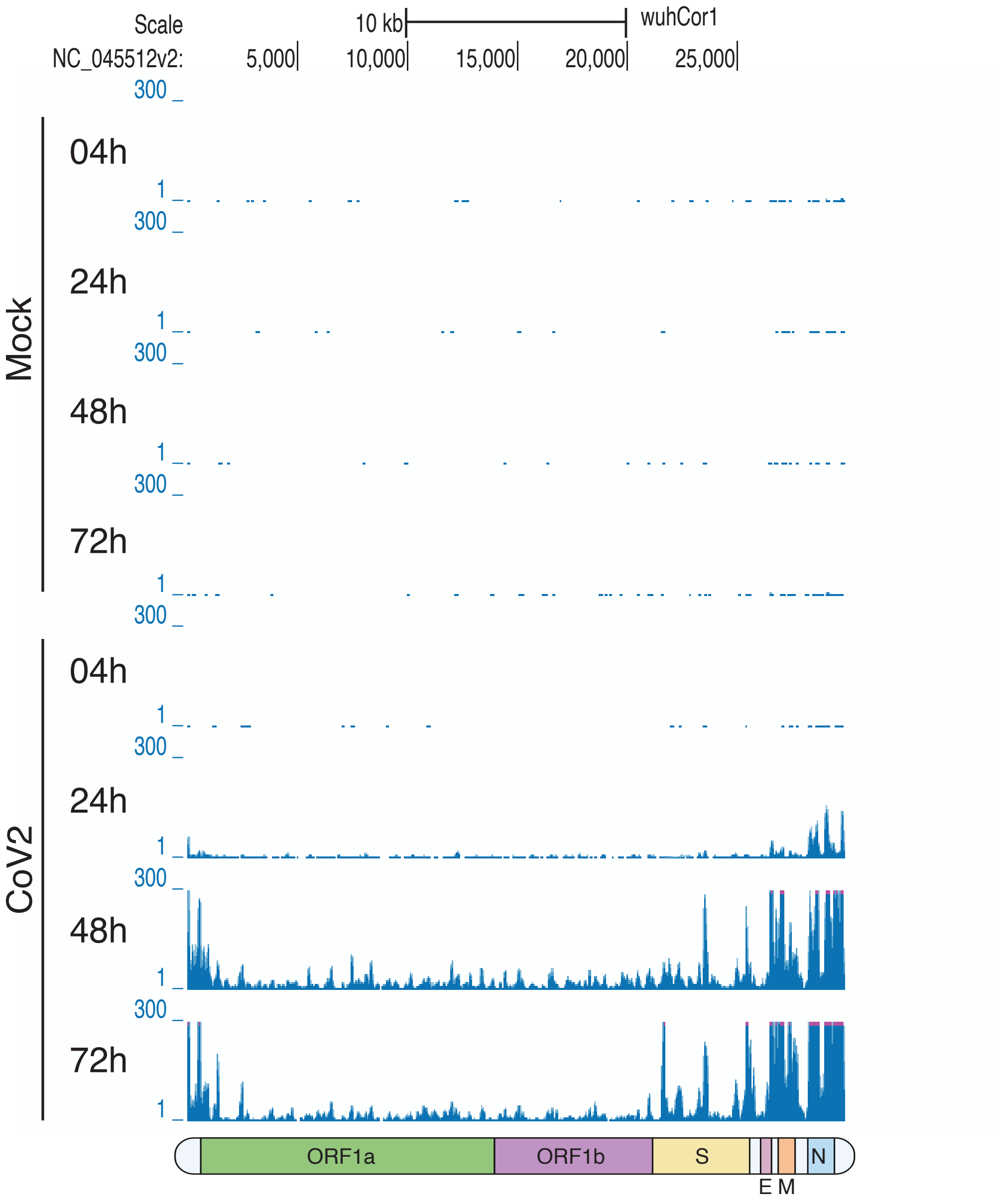

Supplemental Figure 3. sRNAs derived from Vero-E6 cells map to the SARS-CoV-2 genome. UCSC Genome Browser screenshots showing Vero-E6 sample-derived sRNA sequencing reads mapped to the SARS-CoV-2 genome for both infected and control cells. Reads from infected cells do not map uniformly to the genome but instead form “pile ups” in particular regions. Underneath the screenshots is a schematic representation of the SARS-CoV-2 genome depicting the regions of ORF1a/1b, the spike protein (S), the envelope protein (E), the membrane protein (M) and the nucleocapsid protein (N).

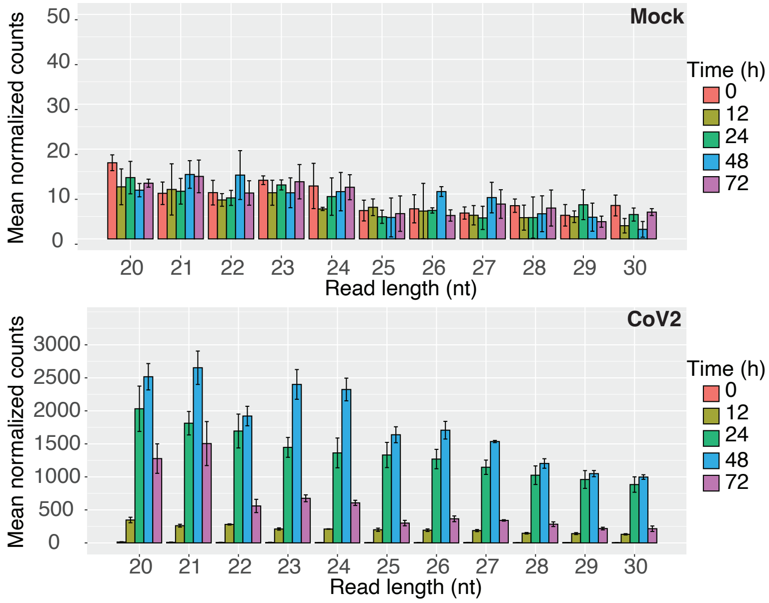

Supplemental Figure 4. Length distribution of Calu-3 sample-derived sRNAs mapped to the SARS-CoV-2 genome. Barplot representation of the length distribution of sRNAs mapping to the SARS-CoV-2 genome for both infected and control cells.

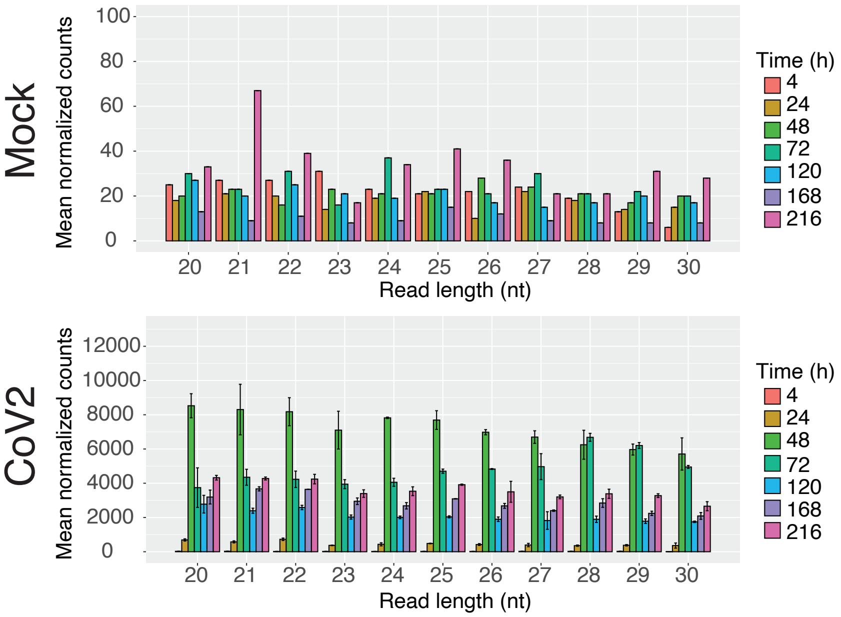

Supplemental Figure 5. Length distribution of Vero-E6 sample-derived sRNAs mapped to the SARS-CoV-2 genome. Barplot representation of the length distribution of sRNAs mapping to the SARS-CoV-2 genome for both infected and control cells.

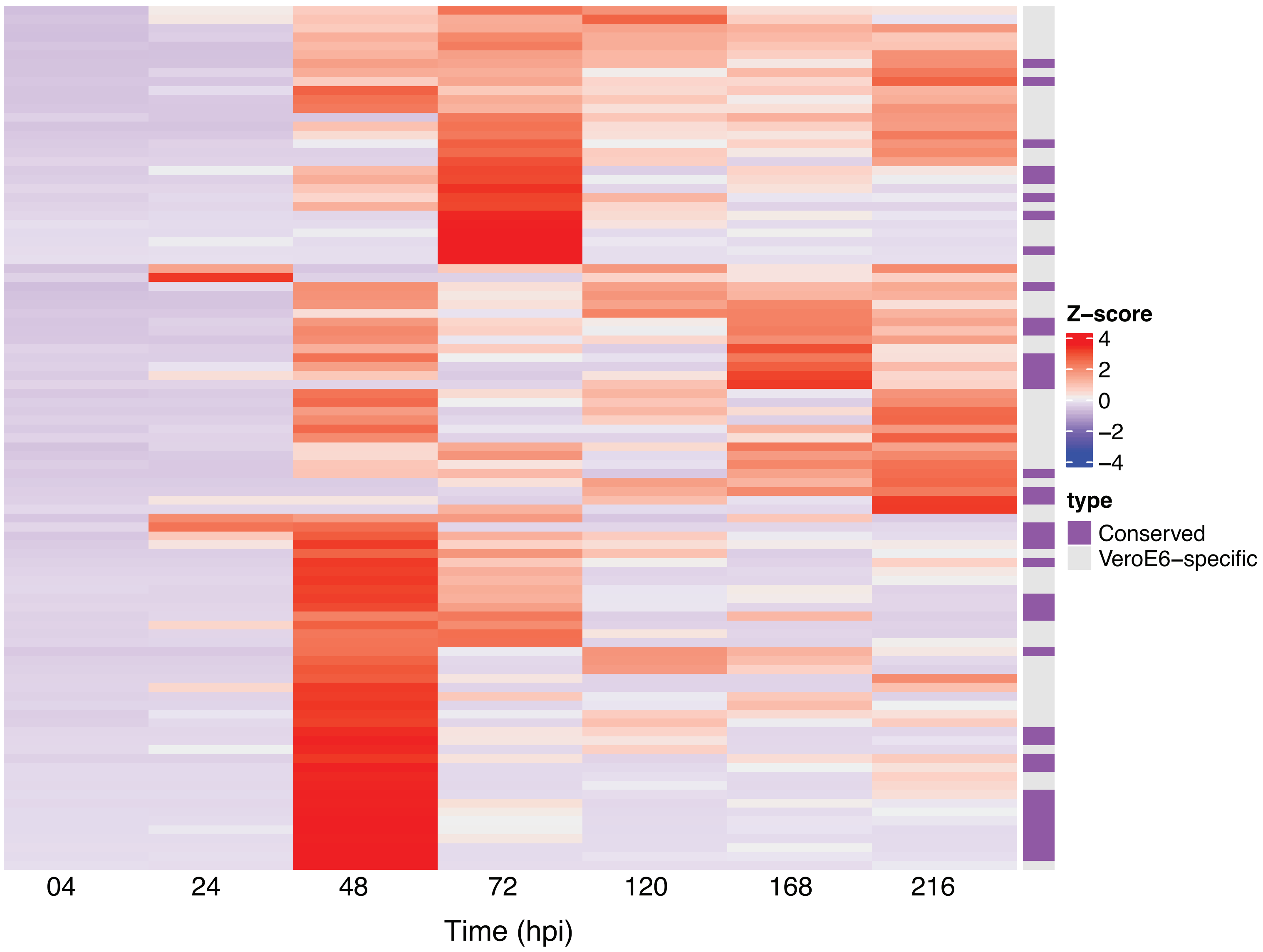

Supplemental Figure 6. SARS-CoV-2 expresses a diverse landscape of small RNAs in infected Vero-E6 cells. Heatmap representation of all differentially expressed svRNAs in Vero-E6 cells. Heat-maps were generated using hierarchical clustering. To the right of the heatmap is an annotation column indicating if the svRNA is species-specific or conserved between Calu-3 and Vero-E6 cells.

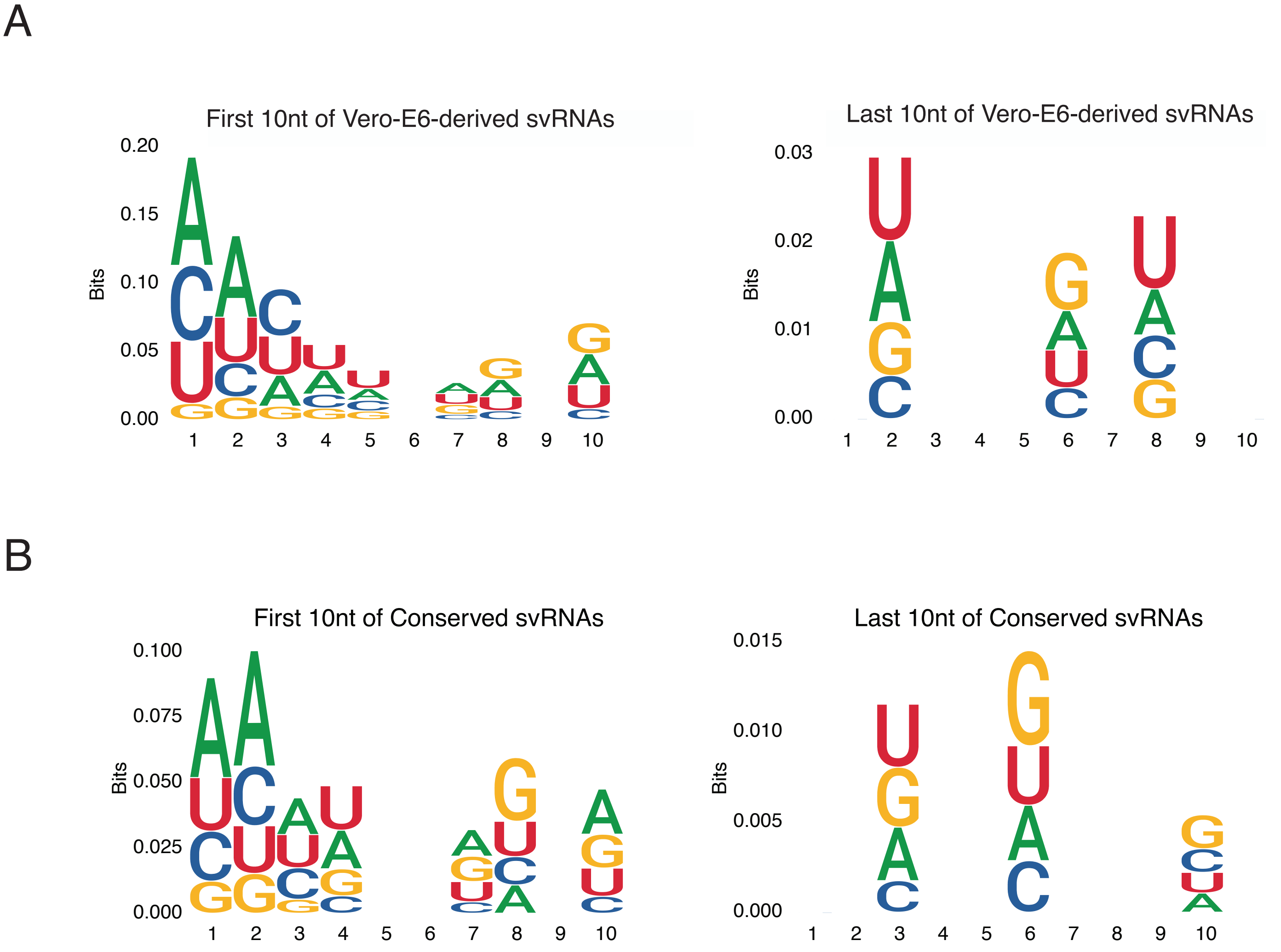

Supplemental Figure 7. Sequence logos of conserved svRNA loci indicate Dicer-independent processing. A. Sequence logos for the first ten and last ten nucleotides of the svRNAs identified in Vero-E6. B. Sequence logos for the first ten and last ten nucleotides of the conserved svRNA loci identified between Calu-3 cells and Vero-E6 cells. svRNAs lack a 5'-U signature typical of many canonical miRNAs.

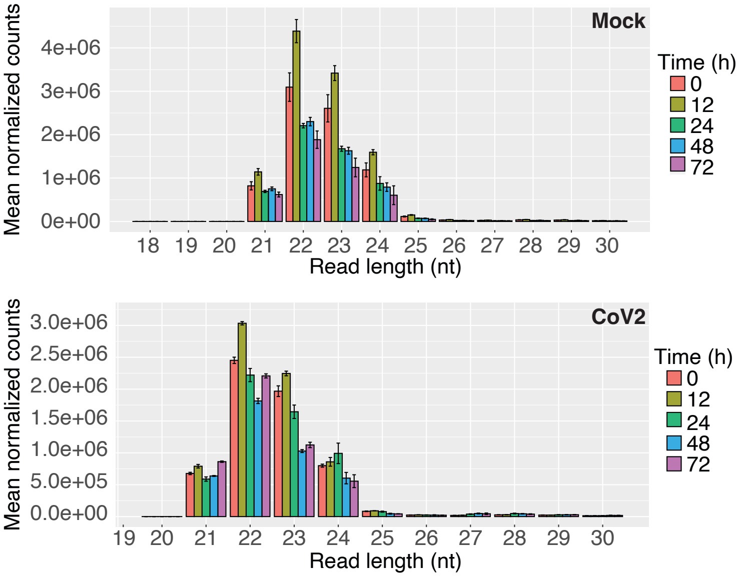

Supplemental Figure 8. Length distribution of Calu-3 sample-derived sRNAs mapped to the GRCh38 (human) genome. Barplot representation of the length distribution of sRNAs mapping to the human genome for both infected and control cells.

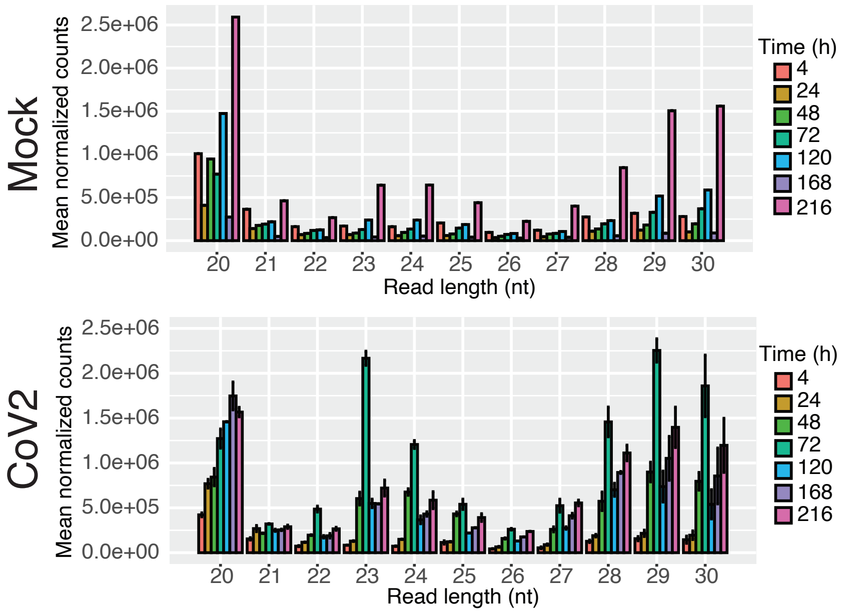

Supplemental Figure 9. Length distribution of Vero-E6 sample-derived sRNAs mapped to the chlSab2 (AGM) genome. Barplot representation of the length distribution of sRNAs mapping to the chlSab2 (AGM) genome for both infected and control cells throughout the time course.

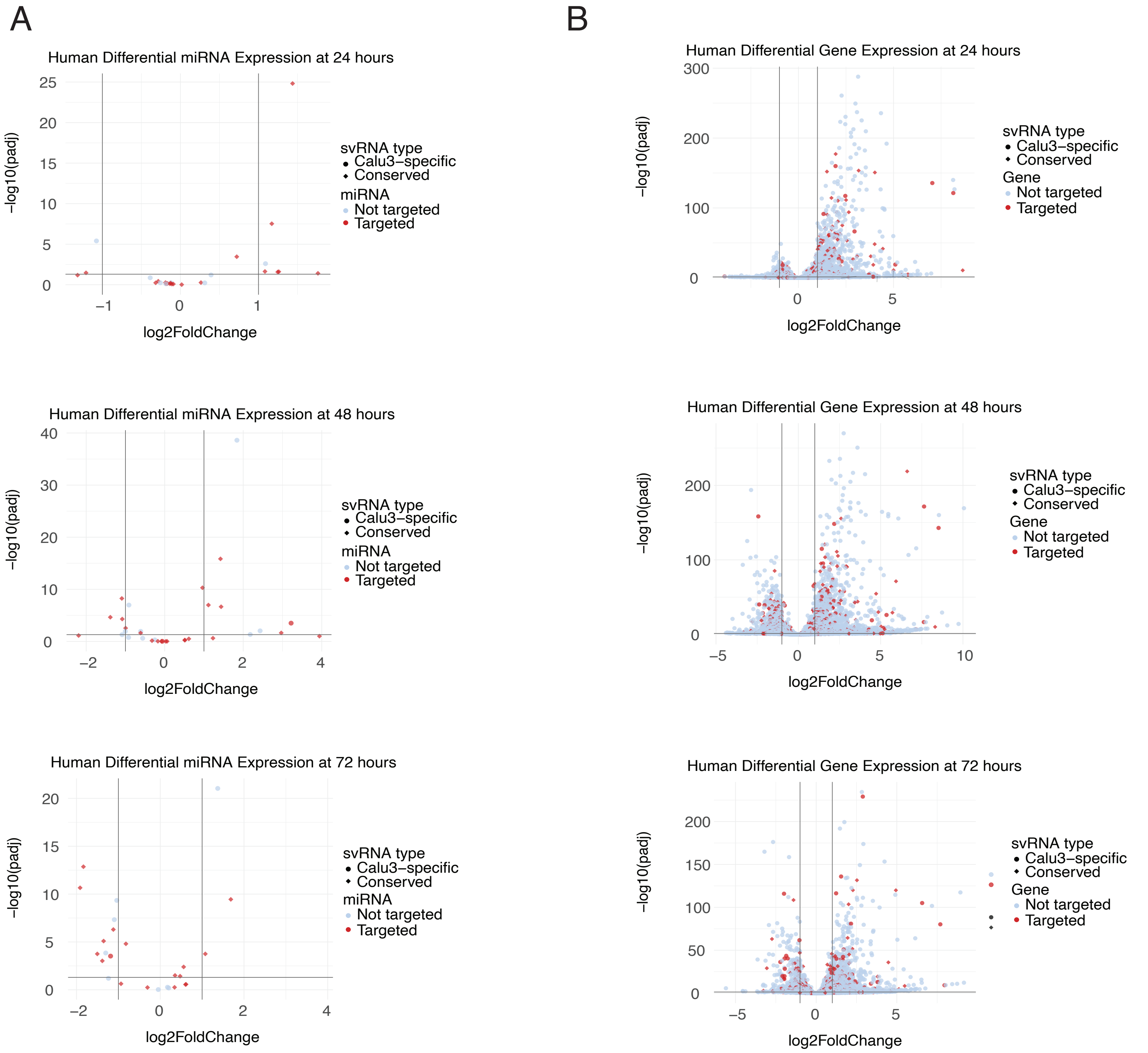

Supplemental Figure 10. Calu-3 sample-derived svRNAs have predicted miRNA and mRNA targets. A. Volcano plots of differentially expressed Calu-3 miRNAs at 24, 48 and 72 hpi. The color of the dots indicates whether the miRNA is a predicted target of an svRNA identified, while the shape of the dots indicates whether a Calu-3 specific or conserved svRNA is predicted to target the miRNA. A significant portion of miRNAs predicted to be svRNA targets fall into the downregulated category within the time course. B. Volcano plots of differentially expressed Calu-3 mRNAs at 24, 48 and 72 hpi. The color of the dots indicates whether the mRNA is a predicted target of an svRNA identified, while the shape of the dots indicates whether a Calu-3 specific or conserved svRNA is predicted to target the mRNA. A smaller portion of mRNAs predicted to be svRNA targets are downregulated within the time course when compared to miRNAs.

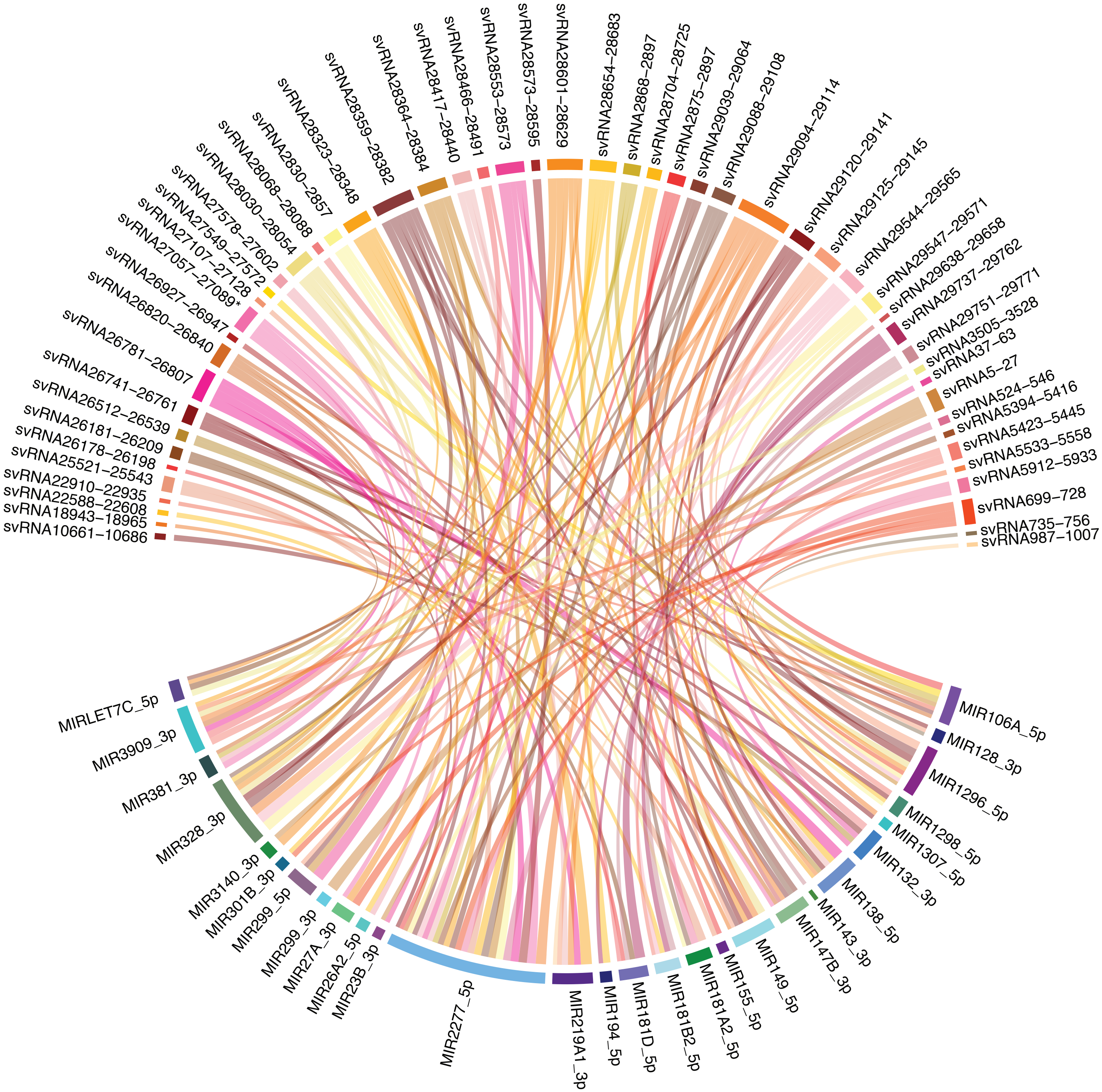

Supplemental Figure 11. svRNAs may target host miRNAs. Circos plot representation of predicted interactions between svRNAs and DE (human) miRNAs using RNAhybrid without seed-forcing. The ribbons are colored according to the svRNA that is predicted to interact with the miRNA to which it connects. The outer ring is a unique color for each svRNA/miRNA, while the inner ring indicates which svRNA/miRNA with which they are predicted to interact. There are many examples of svRNAs targeting more than one miRNA and also examples of miRNAs being targeted by more than one svRNA.

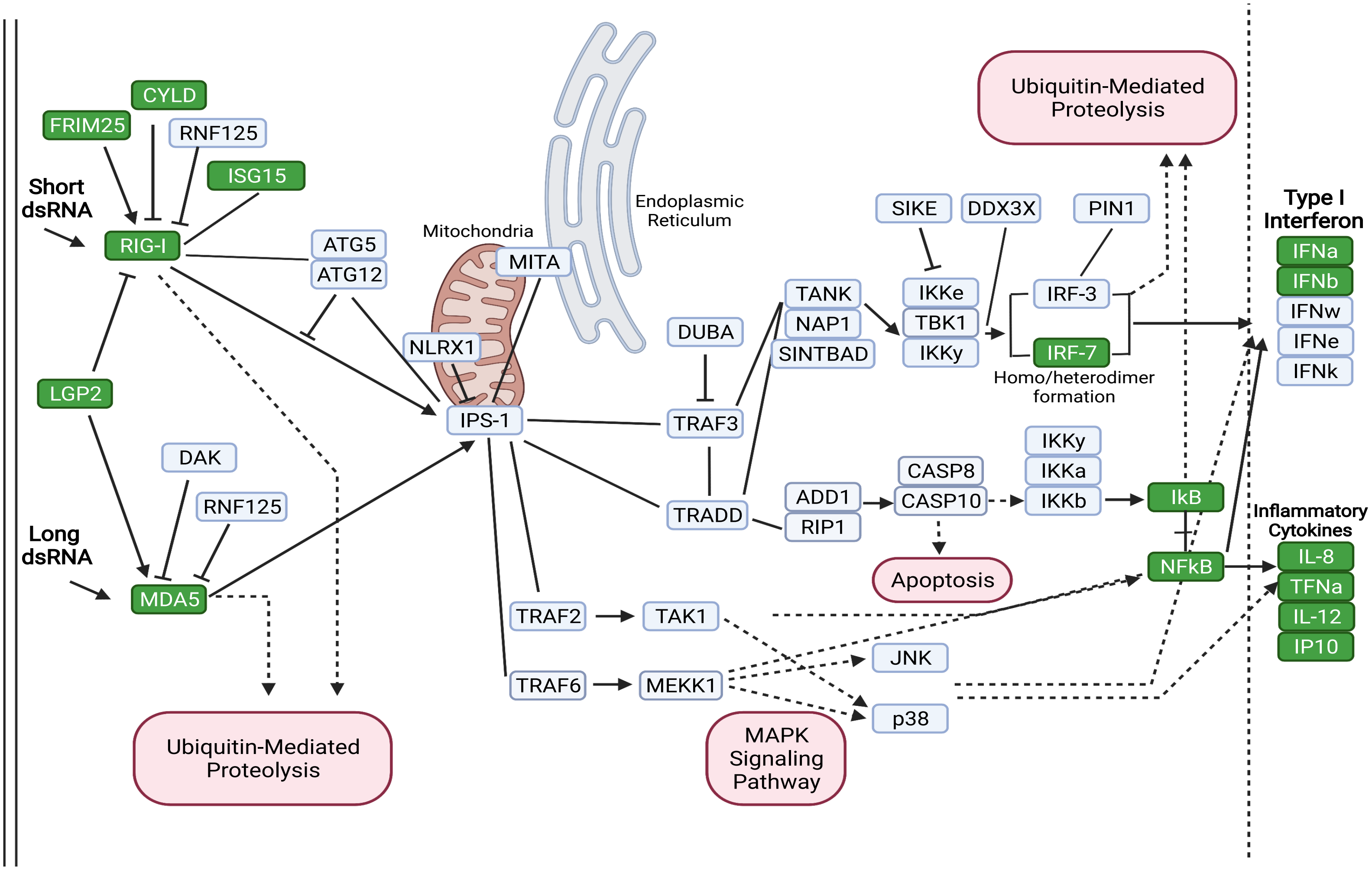

Supplemental Figure 12. Many upregulated Calu-3/Vero-E6 genes are involved in the RIG-I pathway. Schematic representation of the foreign dsRNA-detecting RIG-I pathway. Upregulated genes from our data set are shown in green. Created with BioRender.com.

Name of svRNA	Sequence (5'⟶3')
v-miRNA-ORF7a-27409 (Meng et al., 2021)	UCUUGGCACUGAUAACACUG
svRNA27406-27428	UUUUCUUGGCACUGAUAACACU
v-miRNA-N-28612 (Meng et al., 2021)	AGGAACUGGGCCAGAAGCUGG
svRNA28601-28629	UCUACUACCUAGGAACUGGGCCAGAAGC
v-miRNA-N-29094 (Meng et al., 2021)	UCGGCAGACGUGGUCCAGAA
svRNA29088-29108	AGCUUUCGGCAGACGUGGUC
svRNA29094-29114	UCGGCAGACGUGGUCCAGAAC
svRNA-5p (Cheng et al., 2021)	AAGAUCUCAGUCCAAGAUGGU
svRNA28573-28595	AUGAAAGAUCUCAGUCCAAGAU
svRNA-3p (Cheng et al., 2021)	AGGAACUGGGCCAGAAGCUGGA
svRNA28601-28629	UCUACUACCUAGGAACUGGGCCAGAAGC
vmir-5p (Pawlica et al., 2021)	UUCUUGGCACUGAUAACACUCG
svRNA27406-27428	UUUUCUUGGCACUGAUAACACU

Supplemental Figure 13. svRNAs identified overlap with previously reported svRNAs. Examples of sequence alignments of svRNAs identified in this study and other svRNAs recently reported in the literature.

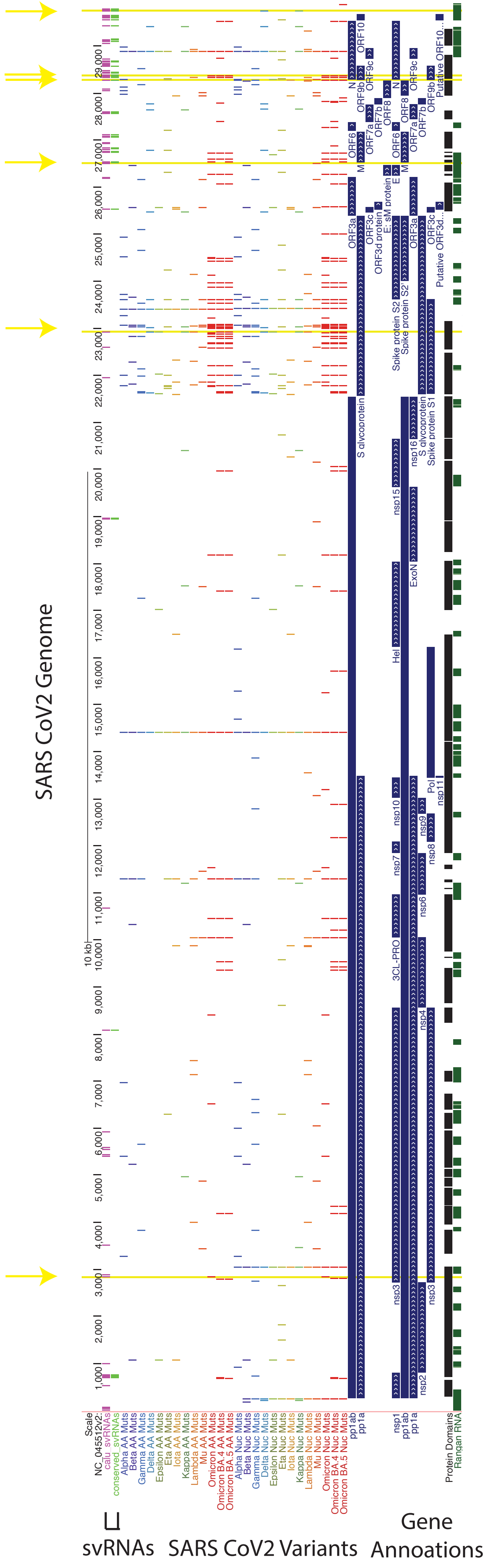

Supplemental Figure 14. UCSC Genome Browser snapshot of SARS CoV2 genome and variants (strains), Calu- and conserved svRNAs (purple and green, respectively).
